# Tobacco Smoke: Involvement of Reactive Oxygen Species and Stable Free Radicals in Mechanisms of Oxidative Damage, Carcinogenesis and Synergistic Effects with Other Respirable Particles

**DOI:** 10.3390/ijerph6020445

**Published:** 2009-02-02

**Authors:** Athanasios Valavanidis, Thomais Vlachogianni, Konstantinos Fiotakis

**Affiliations:** Department of Chemistry, Free Radical Research Group, University of Athens, University Campus Zografou, 15784 Athens, Greece

**Keywords:** Tobacco smoke, free radicals, reactive oxygen species, oxidative stress, mechanisms of carcinogenicity, synergistic effects

## Abstract

Tobacco smoke contains many toxic, carcinogenic and mutagenic chemicals, as well as stable and unstable free radicals and reactive oxygen species (ROS) in the particulate and the gas phase with the potential for biological oxidative damage. Epidemiological evidence established that smoking is one of the most important extrinsic factor of premature morbidity and mortality. The objective of this study was to investigate oxidative and carcinogenic mechanisms of tobacco and synergistic action with other respirable particles in the respiratory system of smokers. Electron Paramagnetic Resonance (EPR) and spin-trapping techniques were used to study stable free radicals in the cigarette tar, and unstable superoxide anion (O_2_^•−^) and hydroxyl (HO^•^) radicals in the smoke Results showed that the semiquinone radical system has the potential for redox recycling and oxidative action. Further, results proved that aqueous cigarette tar (ACT) solutions can generate adducts with DNA nucleobases, particularly the mutagenic 8-hydroxy-2’-deoxyguanosine (a biomarker for carcinogenesis). Also, we observed synergistic effects in the generation of HO^•^, through the Fenton reaction, with environmental respirable particles (asbestos fibres, coal dust, etc.) and ambient particulate matter (PM), such as PM_10_, PM_2.5_ and diesel exhaust particles (DEP). The highest synergistic effects was observed with the asbestos fibres (freshly grounded), PM_2.5_ and DEP. Finally, we discuss results from our previous study of conventional cellulose acetate filters and “bio-filters” with hemoglobin impregnated activated carbon, which showed that these filters do not substantially alter the free radical content of smoke in the particulate and in the gaseous phase.

## 1. Introduction

Epidemiological evidence has established that smoking is one of the most important environmental causes of human mortality and morbidity. Tobacco smoking is currently responsible for approximately 30% of all cancer deaths in developed countries [1]. In addition, smoking causes greater number of deaths from cardiovascular, chronic obstructive pulmonary and degenerative diseases. In 2000, 4.8 million premature deaths worldwide were attributed to smoking, of which 2.4 million in developing and 2.43 million in developed industrialized countries [2], numbers expected to increase to 10 million a year by 2030 [3].

Tobacco smoke is an aerosol containing about 10^10^ particles/mL, consisting of highly porous carbonaceous polymeric material with adsorbed heavy metals, polycyclic aromatic hydrocarbons (PAH), azaarenes, N-nitrosamines and various other organic chemicals. The particular phase of tobacco smoke contains at least 3,500 chemical compounds and a high proportion of them are toxic, carcinogens or mutagens, (e.g. benzene, 2-napthylamine, ^210^Po, ^226^Ra, ^228^Ra, nickel, cadmium, benzo[a]pyrene, etc) [4]. There are at least 55 carcinogens in cigarette smoke that have been evaluated by the International Agency for Research on Cancer (IARC) with “sufficient evidence for carcinogenicity” [5, 6].

Tobacco smoke is divided into the mainstream (smoker inhaled) and the sidestream smoke. The mainstream is divided into a particulate solid phase (tar) and the gas phase (toxic gases, volatile organic compounds, VOCs, free radicals, etc.). Cigarette tar contains remarkably high concentrations of stable free radicals (ca. 10^17^ spins·g^−1^) with very long lifetimes. The sidestream smoke is divided in the solid and gas phases, containing higher concentrations of toxic and carcinogenic compounds and other volatile and semivolatile compounds [7–9]. Free radicals and oxidants in the gas phase exist in a steady state in which they are continuously formed and destroyed and their concentration increases as the smoke ages [10, 11]. The gas phase is around 0.4–0.5 g/cigarette and contains ca. 500 volatile organic and inorganic compounds [12]. The particulate phase (tar) consists of fine and very fine particles (0.1–1.0 μm, aerodynamic diameter) penetrating deep into the alveoli. Some of the water-soluble components of aqueous cigarette tar (ACT) can produce superoxide anion (O_2_^•−^) and subsequently H_2_O_2_ and the reactive hydroxyl radical (HO^•^), which cause oxidative damage to cellular membrane lipids, proteins, enzymes and DNA [13, 14]. The sidestream smoke consists of similar chemical components in the solid and gas phases and is also rich in highly reactive and short-lived free radicals. Passive smoking (or environmental tobacco smoke, ETS) has been proved to be a health hazard for non-smokers and its burden of major lung diseases [15–17]

Biochemical mechanisms of carcinogenic action by tobacco smoke constituents and oxidative damage in cellular DNA by ROS have been observed with very sensitive techniques [18–20]. In the last two decades, the central role of free radical mechanisms in tobacco smoke carcinogenesis and oxidative stress has been established by a series of studies [21–23]. An important finding by Pryor and co-workers was that cigarette tar has high concentrations of stable free radicals, identified as a semiquinone (QH^•^) and carbon-centered radicals (-C^•^) by EPR [24]. The most interesting is a quinone/semiquinone/hydroquinone (Q/QH^•^/QH_2_) system in the tar polymeric matrix [25]. The stable free radicals were identified as o- and p-benzosemiquinone radicals and the role in the DNA damage, through the formation of HO^•^ were detected by EPR spin-trapping [26]. The following mechanisms have been identified: the QH^•^ radicals reduces O_2_ into O_2_^•−^, which can dismutate to form H_2_O_2_ and then with Fe^2+^ (cigarette tar itself contains mainly high concentrations of iron) can generate through the Fenton reaction highly oxidizing hydroxyl radicals:

(1) $$\text{Q}+{\text{QH}}_{2}⇆2\;{\text{QH}}^{•}$$

(2) $${\text{QH}}^{•}+{\text{O}}_{2}\to \text{Q}+{\text{O}}_{2}^{•-}+\text{H}+$$

(3) $${\text{O}}_{2}^{•-}+2{\text{H}}^{+}\to {\text{H}}_{2}{\text{O}}_{2}$$

(4) $${\text{QH}}_{2}+{\text{O}}_{2}\to {\text{H}}_{2}{\text{O}}_{2}+\text{Q}$$

(5) $${\text{QH}}_{2}+{\text{O}}_{2}\to {\text{O}}_{2}^{•-}+{\text{QH}}^{•}+{\text{H}}^{+}$$

(6) $${\text{Fe}}^{2+}+{\text{H}}_{2}{\text{O}}_{2}\to {\text{Fe}}^{3+}+{\text{HO}}^{•}+{\text{HO}}^{-}$$

Cigarette tar can produce large amounts of H_2_O_2_ in aqueous extracts [27] and oxidants in tar and gas-phase have been implicated in the release of iron from the endogenous enzyme ferritin and alter iron metabolism in the lungs [28, 29]. Oxidants and free radicals in cigarette smoke have been considered as a potential mechanism by which smoking can promote lipid peroxidation of cellular membrane lipids, thus promoting atherosclerosis, endothelial dysfunction and acute clinical events, and increase the risk for cardiovascular diseases [30–32]. ROS in the cigarette gas-phase promote the destruction of endogenous antioxidants (vitamins and enzymatic antioxidants) reducing the vital role of cellular antioxidant defenses [33]. Several studies show that antioxidant vitamins are lower in smokers resulting in systemic oxidative stress [34, 35], whereas dietary antioxidant supplements provide only limited protection to smokers [36, 37].

Studies showed that the synergistic interaction of tobacco smoke with various respirable mineral fibres and fine particular matter (soot, fine dusts) in occupational environments explain the increased lung cancer risks and other occupational pulmonary diseases in industrial workers [38, 39]. The synergistic interaction of ROS from cigarette smoke with asbestos fibres contributes to the increases of lung cancer in workers of asbestos mines [40, 41]. Studies showed that cigarette smoke and fresh grinding of asbestos fibres increased by 2–3 times the production of HO^•^ [42, 43]. The synergistic effects of cigarette smoke and asbestos fibres increased DNA damage in bronchial epithelial cells [45].

Similar synergistic interactions of tobacco smoke free radicals and other carcinogenic agents in occupational environments were observed with radon [46, 47], coal dusts [48] and heavy metals (nickel, chromium, cadmium) in mining occupations [49–52]. Synergy mechanisms between tobacco smoke free radical and heavy metals increases malignant neoplasms in occupationally exposed workers [53, 54]. Studies showed synergy between tobacco smoke, alcohol consumption and occupational exposure to mineral particles increase oral and pharyngeal cancers [55, 56].

The gas phase of tobacco smoke contains a large amount of free radicals (estimated at ∼1 X 10^15^ radicals per puff) [7]. Nitric oxide (NO^•^) is a species of considerable interest because of its multiple physiological role (neurotransmission, blood pressure modulator) [57], but also for its toxic effect when generated in excess. NO^•^ reacts quickly with O_2_^•−^ to form peroxynitrite (O=NNO^−^), a chemical known for its highly toxic and oxidative action towards biomolecules (e.g. 3-nitrotyrosine) [58, 59]. NO has been implicated in carcinogenesis and tumour promotion [60].

In the present study reported here we have investigated: first the presence of stable free radicals in the tar of the mainstream cigarette smoke in three different cigarettes (low, middle and high tar content) with conventional acetate filters, by EPR. Secondly, the production of reactive ROS and especially HO^•^ from aqueous tar extracts at physiological pH. Thirdly, we studied the spin-trapping of free radicals in the gas-phase of the sidestream smoke. Fourthly, the production of the hydroxyl adduct to DNA nucleobases, such as the mutagenic 8-hydroxy-2’-deoxyguanosine in aqueous buffered solutions. Furthermore, we present results from the synergistic production of HO^•^ from the interaction of cigarette tar and five different types of fibers, dusts and particulate matter of environmental importance (asbestos fibers, coal dusts, talc dust, PM_10_ and PM_2.5_). Finally, we discuss the results of a previous study which investigated the claims that the so called “bio-filters” (containing dry hemoglobin impregnated in activated carbon) can reduce oxidants, free radicals and carcinogens in mainstream cigarette smoke.

## 2. Materials and Methods

### 2.1. Chemicals

The spin-traps 5,5-dimethyl-1-pyrroline-*N*-oxide (DMPO) and phenyl-*tert*-butyl nitrone (PBN) and the stable 2,2-diphenylpicrylhydrazyl free radical (DPPH, 95%) were purchased from Sigma-Aldrich. 2’-Deoxyguanosine and standard 8-hydroxy-2’-deoxyguanosine (8-OHdG), ethylenediamine tetraacetic acid sodium salt (EDTA-Na_2_) and deferoxamine mesylate salt (desferrioxamine) were purchased from Sigma. All other chemicals were from Aldrich, Merck and Fluka.

### 2.2. Cigarette Smoking Methods: The standard puff protocol

**Figure 1.**
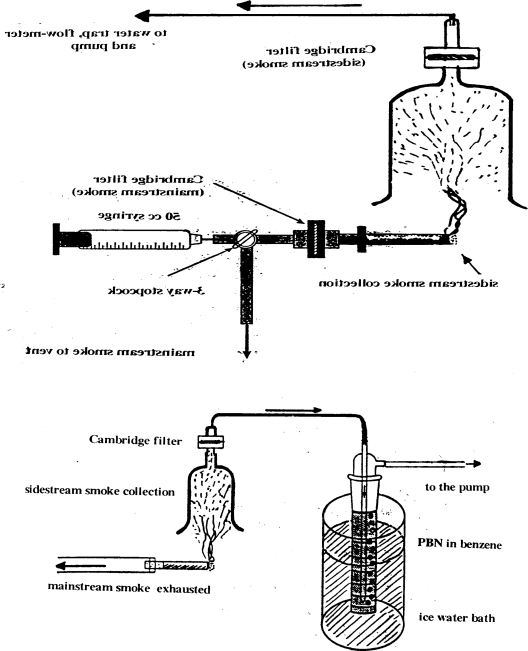
Diagrams of the methods used for the collection of mainstream and sidestream cigarette smoke. The standard puff protocol: a hand held syringe was used to draw puffs of smoke (30 mL puff x 3 sec duration at 1 min. intervals, approx. 10 puffs per cigarette). Tar was collected on Cambridge filters. The second diagram shows the method used for the collection of the tar and gas-phase of sidestream cigarette smoke. The gas-phase (after retaining tar with a filter) was bubbled through 10 mL of the spin-trap PBN in benzene.

Puffs of 30 mL were pulled (3 s duration) at 1 (one) min intervals, using a hand-operated 50 mL syringe, until a fixed butt length was reached (∼10 puffs per cigarette) and passed through a Cambridge filter (glass fibre filter retaining 99% of particles larger than 0.1 micron) which collected the tar. Three different cigarettes were used: (1) low tar, ∼3 mg/cigarette, (2) middle tar, ∼8 mg/c, and (3) high tar, ∼14 mg/c. Tar was extracted with 20 mL of benzene (sonication) and the volume was reduced under reduced pressure in a rotavapor to dryness. The tar of the sidestream was collected in the intervals after passing by a second Cambridge filter, but not used in our experiments, since the smoker breaths only a small amount depending on the enclosed space of smoking. In the second method the gas-phase of the cigarette smoke (after passing though a Cambridge filter to retain tar) was bubbled through 10 mL solution of the spin-trap (0.1 M, in benzene) phenyl-*tert*-butyl nitrone, (PBN). In all experiments five cigarettes were smoked for every experimental measurement. Both diagrams of apparatus used in our cigarette smoke collection are shown in Figure 1. A more detailed analysis of their function is explained in detail in a previous paper [61].

### 2.3. Measurements of Stable Free Radical in Cigarette Tar by EPR

Tar collected in the Cambridge filter of the mainstream smoke of five cigarettes (the experimental procedure was the same for all three brands of cigarettes) was extracted with distilled benzene (sonicated for 20 min). The solvent was evaporated to dryness and was dried further under vacuum and under a dry nitrogen stream. The dried residue is a thick dark brown colour tarry substance. Tar was weighed (∼5 mg) inside an EPR quartz tube for spectroscopic measurements. Spectral parameters (Varian E-4, X-band spectrometer with 100 kHz field modulation): microwave power, 10–20 mW; modulation amplitude, 1–1.4 G (Gauss); scan range 100 G; time constant, 1.0 s; scan time, 16 min; receiver gain, 1–8 X 10^3^ (depending on the intensity of the EPR lines). The radical spins/g values of equal amounts of samples of the three brands of cigarette tar were determined by comparison with known amounts of the free radical DPPH. The area under the EPR single-broad peak was cut carefully and the paper was weighed, representing a quantitative measure of the concentration of radical spins per unit of weight (1 g).

### 2.4. Spin-Trapping and Measurements of *O_2_^•−^* *and HO^•^* *Generated by* Aqueous Cigarette Tar (ACT) Solutions at Physiological pH

Experiments for 50 mg of dried cigarette tar residue was added in 10 mL phosphate buffered solutions (pH =7.4) and 1 mL of 0.08 M aqueous DMPO spin-trap was added. The mixture was shaken gently by a mechanical shaker in the dark (covered with aluminum foil), at room temperature for 2–3 min and placed in an EPR quartz flat cell prior to recording its EPR spectrum. Spectral parameters (Varian E-4, X-band spectrometer with 100 kHz field modulation): microwave power, 20 mW; modulation amplitude, 1.0 G; scan range 100 G; time constant, 1.0 s; scan time, 16 min; receiver gain, 5–8 X 10^3^. The 6-line EPR spectrum of spin DMPO-OOH of O_2_^•−^ spin adduct was recorded in less than 8 min after mixing (time is crucial because of the instability of the EPR spin adduct of the O_2_^•−^which reverts to the hydroxyl spin adduct). The same experiment can be repeated, but with 15 min shaking and degassing with dry nitrogen under the same conditions of spectral parameters. The 4-line EPR spectrum (1:2:2:1 quartet pattern) EPR spectrum of DMPO-OH for the HO^•^ spin adduct was recorded after 20 min in a EPR quartz flat cell. Complimentary experiments were carried out with ACT in the presence of 0.001 M H_2_O_2_ and in the presence of 0.02 M of the chelating agent EDTA-Na_2_ in order to test the increase of HO^•^, through the Fenton reaction by the Fe(II) ions in the tar. EPR spectra of the DMPO-OH adduct were recorded 15 min after mixing and degassed with dry nitrogen. Addition in the ACT mixture of desferrioxamine chelating agent suppress the formation of HO^•^. All measurements were repeated in triplicate. These EPR spectra are presented in Figure 3.

### 2.5. Spin-Trapping Measurements of the Gaseous Phase of Mainstream Smoke

Five cigarettes were smoked by the continuous flow system and the mainstream smoke, after passing the Cambridge filter, was bubbled through a glass tube containing 0.1 M of the spin-trap PBN in benzene (Figure 1). EPR spectra were recorded by the use of an EPR quartz flat cell and EPR spectral parameters were regulated to give the best resolved spectra of the spin adduct. All experiments were repeated in triplicate.

### 2.6. HPLC/UV-EC Quantitative Measurements of the 8-OHdG from Mixtures of ACT with 2’-dG in Buffered Solutions

Samples were prepared by mixing cigarette tar (50 mg) in phosphate buffered solution (pH 7.4, 10 mL) with 0.05 M 2’-deoxyguanosine (dG, 1 mL). The mixture was shaken gently in the dark for 1 hr at room temperature. The mixture was shaken gently in the dark for 1 hr at room temperature. The mixture was filtered (Gelman Acrodisc 0.2 μm). A control containing only dG was analysed in parallel with the reaction mixture. HPLC (Hewlett-Packard, Agilent 1100), reverse phase-HPLC, Column Lichro (250 x 4 mm), Lichorospher 100 RP-18, 5 μm column (25 cm X 4.6 mm), under isocratic conditions. The mobile phase was 7% methanol-93% buffer solution KH2PO4, 50 mM (pH 7.4) with a flow rate of 1.2 mL/min. The 8-OHdG and dG were monitored at 254 nm, and by an electrochemical detector (Coulotherm II EC, ESA, Inc, Chelmsford, MA) set at 400 mV and 20 nA full scale. Standards of 2’-dG and 8-OHdG help for the calibration. Detection limit of 8-OHdG estimated to 2–3 μM. Each reaction was repeated three times, values, average of 3 trials ± S.D.

### 2.7. Synergistic Effect of ACT with Various Fibres and Respirable Particles

50 mg of dried mainstream cigarette tar were dissolved in 20 mL aqueous phosphate buffered solution (physiological pH 7.4). To the solution was added 50 mg of asbestos (separately for crocidolite and chrysotile). Also, experiments were repeated with other respirable particles, such as coal dust, talc dust [Mg_3_(OH)_2_Si_4_O_10_], airborne particulate matter PM_10_ and PM_2.5_ and diesel exhaust particles (DEP).. One (1) mL of 0.08 M of spin-trap DMPO was added in the mixture and the flask was shaken gently for 10 min. In the case of asbestos fibres, the reaction mixture is a suspension of freshly ground asbestos fibres. Aliquots of the suspension were withdrawn at 20 min, filtered and the clear solution was transferred to an EPR quartz flat cell and the EPR spectrum was recorded. EPR instrument parameters: microwave power, 20 mW; scan range 100 G; modulation amplitude 1.0 G, receiver gain 2.5–8 X10^3^; time constant, 0.1 s; scan time 8 or 16 min. Representative EPR spectra in Figure 4.

### 2.8. Comparison of Conventional Cellulose Acetate Filters and “Bio-Filters” (these experimental results were presented in our previous paper) [61]

Cigarette with conventional acetate filters were compared with cigarettes with “bio-filters”. The standard puff protocol and the continuous flow system were used to compare mainstream cigarette tar (trapped on Cambridge filters) and the gas-phase sidestream. The “bio-filter” (BF-filter) used was from a SEKAP (Greek tobacco company) cigarette, and is an extra filter containing activated carbon impregnated with dry hemoglobin, and advertised in 1994–1995 as a filter that reduces considerably free radicals, reactive oxidants, volatile carcinogens, etc [61, 62].

## 3. Results and Discussion

Cigarette tar is a carbonaceous tarry substance with mainly long-lived radicals, but also contains adsorbed heavy metals and carcinogenic organic compounds in the porous polymeric matrix. Whereas the gas-phase is a mixture of oxidative gases, VOCs and a great variety of small reactive radicals, mainly carbon- and oxygen-centered radicals, of short lifetimes [63, 64].

**Figure 2.**
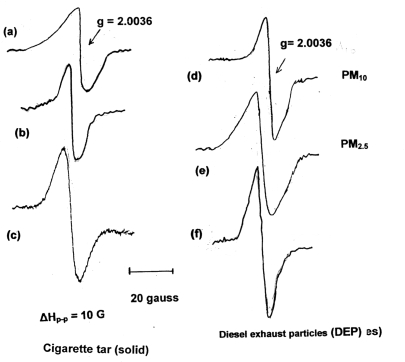
Electron Paramagnetic Resonance (EPR) spectra for the stable free radicals of cigarette tar: (a) cigarette with low tar content, (b) middle tar content, (c) high tar content. The following EPR spectra are representative of ambient airborne particulate matter (PM) in the urban environment: (d) PM_10_, (e) PM_2.5_, (f) Diesel exhaust particles (DEP).

In the first series of experimental observations we focused on the stable free radicals in the cigarette tar of the mainstream. The three types of cigarette all showed a single broad EPR signal with *g* value of 2.0035 (*g*, spectroscopic spitting factor), which has been assigned to the semiquinone radical system. The g value is typical of organic semiquinone (QH^•^) radicals previously observed in aqueous solutions at pH 8.0 [65]. Results are presented in Figure 2.

Comparison of cigarette tar for the three different types of cigarettes showed that the lower tar content the smaller the EPR signal. Although the quantitative comparison is of limited accuracy (estimated to 85–90%), the concentrations of spins/g of tar (using the free radical DPPH as a standard) were estimated as: a. low tar cigarette: ∼10^15^ spins/g; b. middle tar,10^15^–10^16^ spins/g; c. high tar, 10^16^–10^17^ spins/g. Similar results were reported by Pryor *et al*. [66]. Additionally, we compared cigarette tar with similar EPR spectra of particulate matter of atmospheric pollution in urban areas (diesel exhaust soot, PM_10_ and PM_2.5_ collected in the center of Athens, Greece). Diesel exhaust particles (DEP) showed concentrations in the range 10^16^–10^17^ spins/g, which is in the same range with the high tar cigarettes. It is well known that both contain fine and superfine carbonaceous particles in a polymeric matrix [67].

The broad single EPR signal of cigarette tar is known to represent at least six different radicals (some not identifiable) [11], the most important being the semiquinone radical system. Washing the cigarette tar with methanol we removed the semiquinone radical system, the remaining tar showed a much narrower single EPR signal with g 2.0028, which represents a stable carbon-centered radical (-C^•^-) embedded in the polymeric matrix. This radical showed lower potential from the production of HO^•^ radicals (results not shown), as was observed by Sagai and co-workers in washed DEP [68]. Thus, it was concluded that the semiquinone radical system is the potential toxic species (with redox potential) in the cigarette tar and in DEP.

The toxicological implications of cigarette tar due to the presence of these stable free radicals are obvious. Fine and superfine tar particles are deposited in the pulmonary alveoli coming into contact with pulmonary fluids that wash over it and extract the water soluble components. Although cigarette tar radicals do not bind to DNA, the semiquinone system reduces oxygen into O_2_^•−^, which in turn is dismutated into H_2_O_2_ and is decomposed by transition metals, such as iron (ferrous) and copper, to form extremely reactive HO^•^ species. These reactions can take place inside the cellular nucleous and HO^•^ can attack DNA to produce large number of modified nucleobases, strand breaks and other DNA lesions and oxidative damage in mammalian cells (mutagenic adduct 8-OHdG, single-strand DNA breaks) [21, 69].

EPR measurements of the generation of O_2_^•−^ and HO^•^ radicals with ACT were performed in phosphate buffer solution at physiological pH (7.4). In the first experiment we mixed the aqueous solutions of tar with the spin-trap DMPO for only 2–3 min and the EPR spectrum was recorded very quickly (within 8 min) because of its short lifetime. The DMPO-OOH adduct of O_2_^•−^ appears, approximately, for 10 min and then is replaced with the DMPO-OH signal of the HO^•^. Some researchers used DMSO as a solvent to stabilize this particular adduct for a longer period of time [68]. But, all researchers consider that the Fenton reaction (especially Fe^2+^) is the crucial mechanisms for oxidative damage and responsible for a substantially increased production of HO^•^,

(7) $${\text{Me}}^{\text{n}+}+{\text{H}}_{2}{\text{O}}_{2}\to {\text{Me}}^{\text{n}+1}+{\text{HO}}^{-}+{\text{HO}}^{•}$$

The significance of the generation of HO^•^ (through the reactions of cigarette tar and other ambient respirable particles) for carcinogenicity and DNA damage due to their highly reactive and oxidizing nature has been emphasized by many research groups [70, 71].

**Figure 3.**
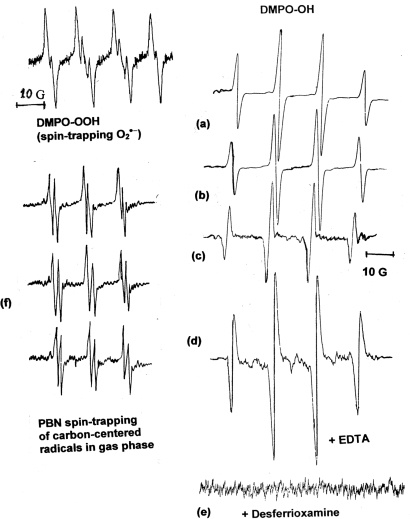
EPR spectra of measurements of and HO^•^ generation from cigarette aqueous tar (CAT) and spin-trapped by DMPO (DMPO-OH 4-line 1:2:2:1): (a) low tar, (b) middle tar, (c) high tar. Representative EPR spectrum of O_2_^•−^ and spin-trapped by DMPO (short-lived, DMPO-OOH adduct). The addition of chelating agents (d) EDTA increases substantially the DMPO-OH radical adduct; (e) desferrioxamine suppresses the radical formation by complexing iron ions. Representative EPR spectra, (f) of the spin adduct with PBN of the gaseous phase organic radical for the three brands of cigarette.

The involvement of metal ions in the generation of HO^•^ radicals has been established by using chelating agents. The addition of the known chelating agent EDTA-Na_2_ in the mixture increases the EPR signal. This is the result of chelation of iron ions, Fe (II), thus lowering their redox potential which is expressed in higher formation of HO^•^. Dalal *et al*. [48] showed that coal mine dust with H_2_O_2_ produces high concentrations of HO^•^ (spin-trapped by DMPO). But the addition of EDTA enhances the HO^•^ generation due to the coal dust surface ferrous ions (blocks all valences minus one). The opposite happens with the chelator desferrioxamine, well known EPR signal of DMPO-OH adduct is suppressed, almost completely with the addition of the chelating agent desferrioxamine (known for chelating all valences of iron, thus reducing drastically its redox potential). These experimental observations suggest the important role of iron and other metals in the oxidant generating activity. These experiments were repeated with the addition of 0.01 M H_2_O_2_. As was expected the EPR signal of DMPO-OH adduct increased substantially. Results of all EPR spectra are presented in Figure 3.

The gaseous phase of mainstream cigarette smoke contains a great variety of gaseous (including CO and NO) and volatile chemicals but also organic free radicals. The use of spin-trap PBN in benzene was used to trap these radicals. The EPR spectra suggest that the principal radical species in cigarette smoke were oxygen- and carbon-centred radicals (-C^•^-, and -O^•^-) with hyperfine splitting factor g= 2.0028–2.0035, and hyperfine splitting constants of a_N_=14.0 G and a_H_=2.0 G. These radicals are difficult to separate, but their structure is probably alkoxyl or/and peroxyl [72]. All types of cigarette gave very similar intensity EPR signals, suggesting that low and high tar cigarettes produce similar gaseous phases.

Incubation of 2’-deoxyguanosine (dG) with ACT resulted in the formation of the mutagenic 8-OHdG (or its stable product 8-oxo-2’-deoxyguanosine, 8-oxodG), which is used for quantitative measurements of oxidative DNA damage and as a biomarker of the initial stages of carcinogenesis [73, 74]. This evidence of oxidative damage in various forms of DNA *in vivo* is very different than “naked” nucleosides, such as dG. But is a representative oxidative damage to cellular DNA which can formed by HO^•^ attacking the electron reach 8-position of the nucleobase guanine. Our results which included the three types of cigarette in phosphate buffer, pH 7.4, the ambient particulate matter (center of Athens) PM_10_, PM_2.5_ and diesel exhaust particles (collected on filters from the exhaust pipe of a diesel car under special conditions) and a mixture of asbestos fibres (freshly grinded) with ACT in the presence of dG, are presented in Table 1.

Results showed that ACT is a powerful oxidant generating HO^•^. The higher the tar content of the cigarette the higher the potential for HO^•^ production, but the differences are relatively small among the three types of cigarettes. The PM in the presence of H_2_O_2_ produces relatively smaller amounts of HO^•^ than ACT. PM_2.5_ and DEP have very similar potential with ACT, possibly because of its hyperfine particles and the adsorbed metal ions (including ferrous ions). The production of HO^•^ by mixtures of asbestos fibres and ACT is doubled under the same conditions indicating synergistic effects. Similar results were published by other studies for inhalable PM, oil and fly ash, coarse and fine PM, focusing on the role of redox active metals and their bioreactivity [75–78].

**Figure 4.**
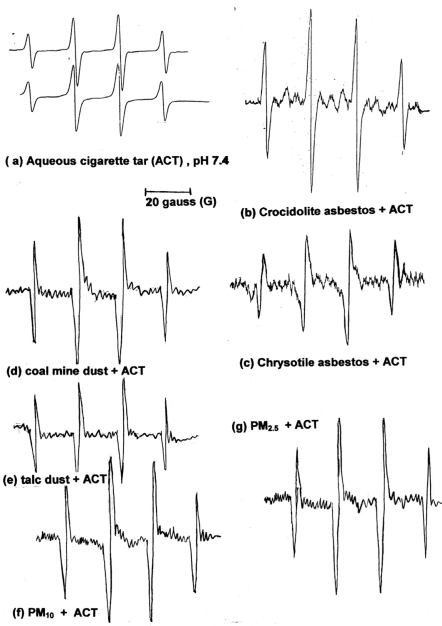
Representative EPR spectra of the synergistic effect of ACT with various respirable particles: (a) only ACT in aqueous buffer (pH 7.4), (b) asbestos fibres (crocidolite) and ACT, (c) asbestos (chrysotile) and ACT, (d) coal mine dust and ACT, (e) talc dust and ACT, (f) PM_10_ and ACT, (g) PM_2.5_ and ACT.

Asbestos fibres (crocidolite and chrysotile), coal mine dust, talc dust, PM10 and PM2.5 with aqueous cigarette tar (ACT) in phosphate buffer mixtures generated increasing amounts of HO^•^. The spin-trapped DMPO-OH adducts were measured quantitatively (4-line EPR signal). The asbestos fibres showed an increase of the EPR signal, which was 2.5–3 times in the relative intensity than that found in the absence of ATC extracts. This is a strong indication for a synergistic action of asbestos and ACT in the production of highly damaging HO^•^. In 1996 we published a detailed analysis of this synergistic effect of asbestos fibres and ACT [79]. Similar results and the importance of iron in the increased production of free radicals by asbestos fibres were published by other researchers [80–82]. All other respirable particles showed similar synergistic effect in mixtures with ACT, the highest effect was by PM_2.5_ and DEP, and the lowest by coal mine dust and talc. Representative EPR spectra (DMPO-OH adduct) of the synergistic effect of ACT and respirable fibres or particles mixtures are presented in Figure 4. These spectra are compared to ACT alone in order to show the increase in hydroxyl generation.

Finally, we would like to comment on experimental results (published in our previous paper [61]) on the influence of special antioxidant filters (the so called “bio-filters”) to reduce the oxidants and free radicals in the mainstream smoke and some gases in the gas-phase of the cigarette smoke. Greek scientists invented and promoted a “biological filter” (1995) containing activated carbon impregnated with dry hemoglobin [62]. A well known Greek tobacco company (SEKAP) used the bio-filter and started a wide public campaign (full page adverts in newspapers, billboard posters, etc) to promote the “protective” effects of the bio-filter. Despite the controversy surrounding their claims of reductions of dangerous chemical constituents (mainly gas-phase chemicals, such as CO and NO) and free radicals, the promotional campaign and the public’s awareness about the health effects of smoking increased the tobacco company’s share of the market by 50% (1997). A study in our laboratory compared the bio-filter cigarette with cigarettes of conventional cellulose acetate filters. Results showed that there were relative small differences, except in the case of CO and other nitrogen oxides. Especially the mainstream cigarette tar of the “bio-filter” was very similar with conventional cigarettes of the same nominal tar content [61]. In the last decade the EU legislation promoted a drastic reduction of the tar content and the abolition of the cultivation of Anatolian type of tobacco in Greece (very high tar content). Greece has the second highest proportion of smokers in Europe (after Cyprus). In our opinion, the introduction of the “bio-filter” gave the smokers the illusion that there are ways to restrict the adverse health effects of smoking. Finally, after five years the advertising and promotion of the “bio-filter” ceased and the cigarette was withdrawn from the market.

## 4. Conclusions

Cigarette smoke is a complex mixture of numerous chemicals with carcinogenic and toxic potential, but also of stable free radicals, reactive oxygen species (ROS) and gaseous free radical species. These chemical species and especially stable semiquinone radicals in tar, have ways to interact with one another and with biopolymers in the smoker’s lungs. The evidence for the significant role of ROS and free radicals in cigarette smoke toxicology has been overwhelming in the last decades. Hydroxyl radical generated by aqueous cigarette tar can cause oxidative DNA damage. Cigarette smoke and respirable fibres and dusts act synergistically in the increasing production of damaging hydroxyl radicals. Filters (so called “bio-filters”) with antioxidant compounds impregnated in active carbon can affect only marginally the composition and toxicity of solid and gaseous phases of cigarette smoke.

## Figures and Tables

**Table 1.** Quantitative HPLC measurements of 8-OHdG formation from 2’-dG by incubation for 1 hr (pH 7.4, at room temperature) with aqueous cigarette tar (ACT). Also, Quantitative measurements with mixtures of ambient particulate matter (PM) and 0.01 M H_2_O_2_ and asbestos fibres with ACT. Units of measurements are μg 8-OHdG per 10^6^ dG.

Material	Mixture	8-OHdG (μg/106 dG)
ACT (low tar)	50 mg	120 ± 10
ACT (middle tar)	50 mg	145 ± 12
ACT (high tar)	50 mg	170 ± 20
PM_10_ + H_2_O_2_	50 mg + 0.001 H_2_O_2_	85 ± 4
PM_2.5_ + H_2_O_2_	50 mg + 0.001 H_2_O_2_	100 ± 6
DEP + H_2_O_2_(diesel exhaust particles)	50 mg + 0.001 H_2_O_2_	115 ± 10
Asbestos fibres (fresh grinding) +ACT	10 mg +50 mg	250 ± 35

* All results are expressed as mean value ± S.D. (n=3).
